# Predictors and outcome of tetanus in newborns in slum areas of Karachi City: a case control study

**DOI:** 10.1186/s13104-015-1301-y

**Published:** 2015-08-07

**Authors:** Arjumand Sohaila, Yasir Shafiq, Shazia Azim, Benazir Baloch, Ali Syed Muhammad Akhtar, Shiyam Sunder Tikmani, Nick Brown

**Affiliations:** Department of Paediatrics and Child Health, Aga Khan University Hospital, Karachi, Pakistan; Department of Paediatrics and Child Health-Research, Aga Khan University Hospital, Karachi, Pakistan; Department of Paediatrics, Salisbury District Hospital, Salisbury, Wiltshire UK; Department of Paediatrics, Aga Khan University Hospital, Karachi, Pakistan

**Keywords:** Tetanus in newborns, Slum, Bin Qasim seaport, Traditional birth attendant

## Abstract

**Background:**

Tetanus in newborns, is an under-reported public health problem and a major cause of mortality in developing countries. This study aimed to determine the predictors and outcome of tetanus in newborn infants in the slums of Bin-Qasim town, Karachi, Pakistan.

**Methods:**

We conducted a case–control study at primary health care centers of slums of Bin-Qasim town, area located adjacent to Bin Qasim seaport in Karachi, from January 2003 to December 2013. Cases were infants aged ≤30 days with tetanus, as defined by the World Health Organization. Controls were newborn infants aged ≤30 days without Tetanus, who were referred for a checkup or minor illnesses. The case to control ratio was 1:2.

**Results:**

We analyzed 26 cases and 52 controls. The case fatality was 70.8%. We identified four independent predictors of Tetanus in newborns: maternal education (only religious education with no formal education OR 51.95; 95% CI 3.69–731), maternal non-vaccination (OR 24.55; 95% CI 1.01–131.77), lack of a skilled birth attendant (OR 44.00; 95% CI 2.30–840.99), and delivery at home (OR 11.54; 95% CI 1.01–131.77).

**Conclusions:**

We identified several potentially modifiable socio-demographic risk factors for Tetanus in newborns, including maternal education and immunization status, birth site, and lack of a skilled birth attendant. Prioritization of these risk factors could be useful for planning preventive and cost-effective measures.

## Background

Elimination of tetanus particularly in newborns is an essential and attainable Millennium Development Goal [1, 2]. Globally, the incidence of tetanus in newborns has significantly fallen and is currently responsible for only 1% of newborn deaths. However, Tetanus in newborns is still a major cause of death in lower and middle income countries, such as Pakistan [3, 4]. In 2008, estimated deaths due to Tetanus in newborns were 59,000 [3] and these mainly occurred in poor communities [3, 4]. Pakistan is one of the 34 countries that have not achieved the global elimination of Tetanus in newborns [5]. According to a WHO study, among 797 reported cases of newborn tetanus in 2005, 518 (65%) were in Pakistan [4]. This, however, is likely to be a substantial underestimate as tetanus remains largely unreported and hidden within the community [4].

To establish predictors and potential strategies to modify the incidence of Tetanus in newborns, we conducted a case–control study in the slums of Bin-Qasim town, Karachi, Pakistan.

## Methods

We conducted a case–control study in primary health care centers located in slum areas of Bin-Qasim town, including Ibrahim Hyderi, Ali Akber Shah Goth, Rehri Goth, Bhains (cattle) Colony, and Bilal Colony from January 2003 to December 2013. The total population of Bin Qasim Town is approximately a million. These areas are located along the Arabian coast adjacent to Bin Qasim seaport in Karachi and have a large community of fishermen living in poverty and unhygienic conditions. Our subjects were newborn infants. Cases were newborn infants aged ≤30 days with Tetanus, as defined by the World Health Organization; either self-referred or referred by community health workers (CHWs) to primary health centers. After documentation and initial treatment, cases were immediately referred from primary health centers to the nearest tertiary care hospital. Controls were also newborn infants aged ≤30 days without Tetanus selected from the same population and referred (by self/CHW) to the same primary health centers, either for a checkup or minor illnesses. The case to control ratio was 1:2. Subject information was collected by the guardians of babies (mostly mothers) at the time of enrollment and, additionally, extracted from health center records. Details include baby’s age and sex, maternal age, parity (either multi or Primi) and educational status etc. Educational status was divided in three categories: None (Neither went to school nor have religious education) went to school (Those completed primary or secondary education), Only religious education (Those who never went to school but had non-formal religious education at home or somewhere else); Vaccination status was defined as vaccinated i.e. complete and un-vaccinated i.e. partial or no vaccination against Tetanus. Delivery site was defined as home or hospital; birth attendant (include skilled birth attendant like doctors or nurses) and Unskilled birth attendant (These are those who had no formal training for safe delivery practices and include relatives, neighbors or Traditional birth attendants (TBA’s) locally known as Dia’s). All the information collected after having informed consent from parents or guardians. This study was approved by the Ethical Review Committee of Aga Khan University Hospital, Karachi.

Data entry and analysis were performed with SPSS 17.0 (SPSS Inc., Chicago, IL, USA). The Chi square test was applied to compare the values of selected variables related to Tetanus in newborn infants. A P value of <0.05 was considered as significant. Associations between candidate predictors of Tetanus were estimated by univariate and multivariate logistic regression, and expressed as odd ratios (ORs) and 95% confidence intervals (CIs).

## Results

Analysis includes 26 cases and 52 controls. The babies were aged from 3 to 16 days of age and there was a male to female ratio of 2:1. Table 1 compares the values of selected variables for Tetanus in newborn infants. Most of the cases (61.5%) were identified in male infants.

**Table 1:** Demographic variables of the population with and without Tetanus in newborns

Risk factors	Tetanus in newborns			
	No		Yes	
	n	%	n	%
Parity				
Uni-para	7	13.5	11	42.3
Multipara	45	86.5	15	57.7
Mother age				
15–25	6	11.5	15	57.7
26–35	36	69.2	9	34.6
36–45	10	19.2	2	7.7
Mother education				
Went to school	34	65.4	2	7.7
None	11	21.2	5	19.2
Only religious	7	13.5	19	73.1
Delivery				
Home	18	34.6	22	84.6
Hospital	34	65.4	4	15.4
Baby gender				
Male	36	69.2	16	61.5
Female	16	30.8	10	38.5
Vaccination status				
Vaccinated	41	78.8	6	23.1
Unvaccinated	11	21.2	20	76.9
Birth attendant				
Unskilled	4	7.7	17	65.4
Skilled	48	92.3	9	34.6

Data adapted from the government sector and a survey by the private sector.

In univariate analysis (Table 2), young maternal age 15–25 years (OR 12.50; CI 2.08–74.80; P = 0.006), multiparity regardless of maternal age (OR 4.71; CI 1.54–14.35; P = 0.006), maternal only religious education with no formal education (OR 46.14; CI 8.69–244.80; P < 0.001), home delivery (OR 10.38; CI 3.10–34.80; P < 0.001), unimmunized maternal status (OR 12.42; CI 4.01–38.43; P < 0.001), and lack of a skilled birth attendant (OR 22.66; CI 6.17–83.27; P < 0.001) at the time of delivery were predictors for Tetanus in young infants. In the adjusted model, significance for maternal age and parity disappeared. Maternal education (only religious education with no formal education; OR 51.95; CI 3.69–73; P = 0.003), vaccination status (unvaccinated; OR 24.55; CI 1.01–131.77; P = 0.011), birth attendant (unskilled; OR 44.00; CI 2.30–840.99; P = 0.012), and delivery site (home; OR 11.54; CI 1.01–131.77; P = 0.049) were robust predictors for Tetanus in young infants (Table 3).

**Table 2:** Univariate logistic regression analysis model predicting risk factors for Tetanus in newborns

Risk factor	Odd ratio	95% confidence intervals		P value
		Upper	Lower	
Maternal age				
15–25	12.500	2.089	74.808	0.006
26–35	1.250	0.232	6.739	0.795
36–45 (reference)				
Parity				
Unipara (reference)				
Multipara	4.714	1.548	14.352	0.006
Maternal education status				
Went to school (reference)				
Only religious	46.143	8.697	244.809	<0.001
None	7.727	1.309	45.601	0.024
Place of delivery				
Home delivery	10.389	3.101	34.800	<0.001
Hospital delivery (reference)				
Gender of babies				
Male (reference)				
Female	1.406	0.525	3.767	0.498
Maternal vaccination status				
Vaccinated (reference)				
Unvaccinated	12.424	4.016	38.433	<0.001
Birth attendant				
Unskilled	22.667	6.170	83.273	<0.001
Skilled (reference)

**Table 3:** Multivariable logistic regression analysis model for predicting risk factors of Tetanus in newborns

General descriptive variables of study areas	Odd ratio	95% confidence intervals		P value
		Upper	Lower	
Maternal education status				
Went to school (reference)				
Only religious	51.954	3.692	731.065	0.003
None	1.620	0.123	21.247	0.713
Place of delivery				
Hospital delivery (reference)				
Home delivery	11.542	1.011	131.774	0.049
Maternal vaccination status				
Vaccinated (reference)				
Unvaccinated	24.559	2.081	289.826	0.011
Birth attendant				
Skilled (reference)				
Unskilled	44.003	2.302	840.998	0.012

Out of 24 cases of Tetanus, 2 patients (8.3%) self- discharged against medical advice (LAMA) and 2 (8.3%) were lost to follow up. The case fatality rate was (70.8%).

## Discussion

In our study, we found that mothers whose infants developed Tetanus were significantly more likely to be unimmunized, have delivered at home and to have been assisted by unskilled birth attendants like relatives, neighbors or traditional birth attendants (TBAs, locally known as dais). Though they have no formal training, dais are part of the community and trusted, therefore, people frequently seek their assistance for delivery at home. Typically, dais do not have clean delivery kits (CDKs) and use whatever is easily available at home for delivery purposes such as a kitchen knife, scissors, and shaving blades. A similar case–control study in Karachi concluded that non-use of a CDK (OR 2.0; 95% CI 1.3–3.1) and lack of a skilled birth attendant (OR 1.7; 95% CI 1.1–2.7) were independently associated with an increased risk for Tetanus in newborn babies [6]. In another study, 80% of Tetanus cases in newborns in Multan were delivered at home (4). In low resource settings, training of TBAs and dais for safe delivery practices and providing them with CDKs for free and in excess appears to be effective strategy for prevention of Tetanus in young infants [6]. In our study, in common with others, lack of maternal immunization against tetanus was also a risk factor for Tetanus in newborn infants [7, 8]. Maternal immunization against tetanus reduces Tetanus associated mortality by 88% (two doses) [9] and by 94% (three doses) [10] in newborns. However in countries, such as Pakistan, trustworthiness and acceptability for recommended vaccines are permanent challenges [11]. Reasons for this include a lack of awareness, illiteracy, and misconception [7]. Similar to other studies, we also observed that maternal only religious education with no formal education [8] and lack of awareness [8] as a predictor for Tetanus in young infants, reflecting misconceptions about immunization. This can be partially overcome by public awareness with frequent visits of female health workers [7], education of TBAs (dais), and use of mass media but many of these beliefs run very deeply.

In our study, case fatality due to Tetanus in newborn infants was 70.8%, which is consistent with other community studies [4]. Although case fatality in such settings is high, up to 78% survival is reported in tertiary care settings with intensive care and respiratory support [4]. Billoo et al. [12] reported reduced mortality from 24 to 50% by active involvement of mothers at the Civil Hospital in Karachi. In another study, Tetanus-related case fatality among newborns was 30.1% [13]. This previous study showed an admission rate of 96% over 11 years and all of them had received standard treatment. The authors discussed that a low case fatality may be due in part to hospitalization bias for severe cases and the infants being discharged on guardian’s request [13]. Improved hospitalization, use of a standard treatment protocol, and good nursing care account for a low case fatality [14, 15].

This study has some limitations including a small sample size, under-reporting or underestimation of cases due to failure of health care professionals to make reports, and residual confounders.

## Conclusions

Although we cannot exclude residual confounding i, we found some modifiable socio-demographic predictors for Tetanus in young infants, including maternal education and immunization, birth site, and birth attendants at the time of delivery in a poor slum setting in Pakistan. Effective preventive strategies include further improvement from the presently existing immunization and health-service infrastructure. Public awareness and health education, maternal vaccinations, and promoting safe delivery practices by formal training of TBAs (dais) should be part of strategic planning. Risk factors that are identified should be given priority for planning of preventive and cost-effective measures. The renewed worldwide commitment for elimination of Tetanus in newborn infants could successfully translated into elimination by the universal introduction of a number of very simple measures.

## Abbreviations

CHW’s: community health workers
TBAs: traditional birth attendants
CDKs: clean delivery kits
